# Coexistence of Bilateral Giant Adrenal Myelolipomas and Congenital Adrenal Hyperplasia: A Case Report

**DOI:** 10.7759/cureus.47266

**Published:** 2023-10-18

**Authors:** Waleed M Almutairi, Nouf Alshamrani, Ahmed R Alibrahim

**Affiliations:** 1 Endocrinology and Metabolism, King Abdulaziz Medical City Riyadh, Riyadh, SAU

**Keywords:** testicular adrenal rest tumors, glucocorticoid therapy, infertility, congenital adrenal hyperplasia, adrenal myelolipoma

## Abstract

Adrenal myelolipomas (AMs) are rare and benign neoplasms, consisting of adipose and mature hematopoietic tissue. They are commonly discovered incidentally with increased use of radiologic imaging. A small number of giant bilateral adrenal masses are reported, particularly in the setting of congenital adrenal hyperplasia (CAH). We report the case of a 36-year-old male with a history of CAH on steroids since childhood, self-discontinued shortly after diagnosis, presenting mainly with abdominal distension and pain besides infertility. Imaging revealed giant bilateral adrenal masses. Subsequently, he underwent bilateral adrenalectomy, and the surgical pathology report revealed myelolipomas measuring 39×17×8 cm on the left and weighing 4050 grams and 28×16×5 cm on the right and weighing 1702 grams. AMs are found to coexist with many other conditions such as Cushing’s syndrome, pheochromocytoma, and CAH. We discuss the association with high adrenocorticotropic hormone (ACTH) states and review the studies involving ACTH as a stimulator leading to myelolipomas. This case report highlights the proper history taking and biochemical evaluation for early detection and intervention to avoid catastrophic consequences.

## Introduction

Adrenal myelolipomas (AMs) are rare, benign tumors composed of mature adipose tissue and hematopoietic elements, most commonly found in the adrenal gland [1]. The tumor is usually non-functioning, asymptomatic, and discovered incidentally in imaging studies. However, in rare cases, AMs may present with symptoms like abdominal pain or early satiety and vomiting due to the mass effect on adjacent organs. AMs can occur unilaterally or bilaterally, and their size can vary greatly, with some reported cases exceeding 30 cm in diameter [2].

In various research studies examining patients with adrenal myelolipomas, a range of findings regarding adrenal hormone excess have been reported. In one investigation involving 126 patients at a single center, it was found that 3% exhibited autonomous cortisol secretion, while 12% were diagnosed with primary aldosteronism [3]. Another study with a smaller cohort of 65 individuals noted a 4.6% rate of adrenal hormone excess, albeit with an incomplete diagnostic process [4]. Furthermore, in a larger sample of 150 patients, a limited subset of 20 individuals were tested for endocrine dysfunction, revealing that autonomous cortisol secretion was present in three patients and one had primary aldosteronism [5].

In addition to these studies, a minor case series highlighted the occurrence of adrenal cortical hyperplasia in patients experiencing excess levels of aldosterone, cortisol, or androgens [6]. Moreover, instances of AMs have been documented in individuals with Carney complex [7].

While exceedingly uncommon, AMs have been associated with several other conditions, including aldosterone-secreting adrenocortical carcinoma [8], pheochromocytoma [9], and congenital adrenal hyperplasia (CAH) [10]. Latter's occurrence owes to an increase in secretion of adrenocorticotropic hormone (ACTH) as a result of decreased production of cortisol, leading to adrenal hyperplasia. It is supported by a high prevalence of adrenal masses in patients with CAH [11]. The coincidence of myelolipoma and congenital adrenal disorder with subsequent overproduction of the ACTH and androgens might be explained by the incipient of myelolipoma via chronic hormonal stimulation of the adrenal gland cortex [10]. We report a case of giant bilateral AMs coexisting with CAH.

## Case presentation

A 36-year-old male with no known past medical or surgical history presented to the emergency room (ER) with a one-day history of severe right flank abdominal pain that is associated with nausea along with loose bowel motion; however, there were no vomiting or urinary symptoms. Systematic review was also unremarkable. The patient reported the same complaints for two years, that is, waxing and waning in nature with 11 kg weight loss over this time. No medical attention was sought for it. 

On examination, he was anxious and in mild-moderate pain and an overweight male (height 163 cm, weight 75.5 kg, BMI 28.4 kg/m²). He was mildly tachycardic (heart rate 118 beats per minute), but his other vital signs were within the normal range. His abdomen was distended and dull upon percussion, and a large mass was palpable over the whole abdomen.

Initial labs showed erythrocytosis (hemoglobin 168 gm/L), hyponatremia (sodium 131 mmol/L), acidosis (carbon dioxide 16 mmol/L), and ketonuria. Other tests like the coagulation profile and comprehensive metabolic panel were not significant (Table 1).

**Table 1:** Laboratory and urine analysis results Hgb: hemoglobin, WBC: white blood cells, PLT: platelet, INR: international normalized ratio, PT: prothrombin time, PTT: partial thromboplastin time, eGFR: estimated glomerular filtration rate, BUN: blood urea nitrogen, Na: Sodium, K: Potassium, CO2: carbon dioxide, Phos: Phosphorus, Adj Ca: adjusted calcium, Glu R: random glucose, ALT: alanine aminotransferase, AST: aspartate aminotransferase, Bili T: total bilirubin, Alk Phos: alkaline phosphatase, UA: urine analysis, Leuk Est: leukocytes esterase, HPF: high-power field.

Exam	Result	Reference Range
Hgb	168	120-160 gm/L
WBC	4.18	4-11 x10^9/L
PLT	178	150-400 x10^9/L
INR	1.16	0.80-0.120
PT	12.8	9.38-12.34
PTT	30.50	24.84-32.96
eGFR	135	>60 mL/min/1.73 m2
Creatinine	62	50-98 umol/L
BUN	3.4	2.5-6.7 mmol/L
Na	131	136-145 mmol/L
K	4.4	3.5-5.1 mmol/L
CO2	16	22-29 mmol/L
Phos	1.23	0.74-1.52 mmol/L
Adj Ca	2.1	2.1-2.55 mmol/L
Glu R	5.4	2.9-7.8 mmol/L
ALT	14	5-55 U/L
AST	19	5-34 U/L
Albumin	45	35-52 g/L
Bili T	23.6	~ 20 umol/L
Alk Phos	58	40-150 u/L
UA Appear	Clear	Clear
UA Color	Light-Amber	Pale-Dark yellow
UA Glucose	Negative	Negative
UA Ketones	80	Negative
UA Blood	0.03	Negative
UA Leuk Est	Negative	Negative
UA WBC	2	0-5 HPF
UA Nitrite	Negative	Negative
UA RBC	2	0-5 HPF

**Figure 1:**
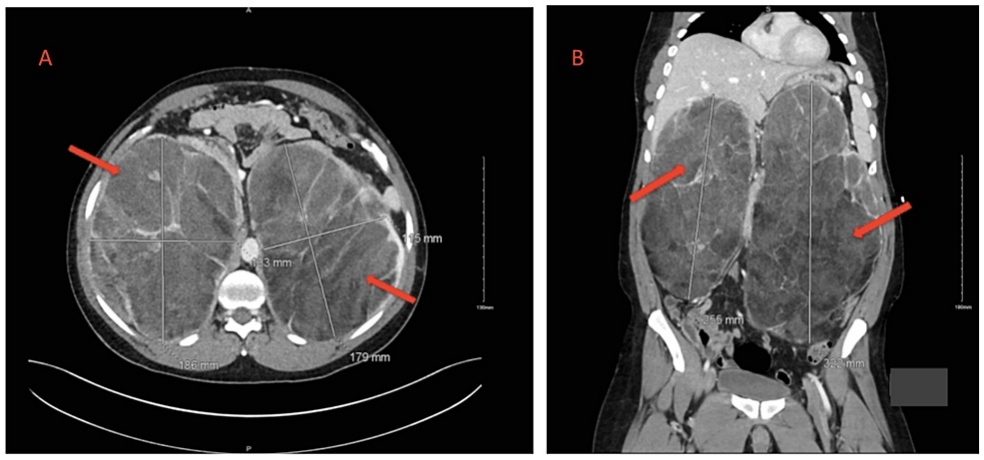
Axial (A) and coronal (B) views of the CT scan demonstrate bilateral adrenal masses (arrows) that are predominantly fatty components interspersed with areas of higher attenuation inside, consistent with giant myelolipomas. CT: Computed tomography.

While in the ER, after a discussion of the risks and benefits, a contrast-enhanced abdomen and pelvis CT scan was done and showed no radiological evidence of appendicitis or nephrolithiasis; however, an abnormal adrenal finding was detected (Figure 1); there were two large well-circumscribed retroperitoneal adrenal masses. The right mass measured 13x18x25 cm while the left mass measured 11.5x18x32 cm. Both masses showed predominantly fatty components interspersed with areas of higher attenuation inside. They are seen on the superior-lateral aspect of both kidneys, the right mass abutting the liver inferiorly, while the mass on the left abutting the pancreas posteriorly. Both kidneys were displaced inferiorly. There was no invasion of surrounding structures.

The patient was admitted under the General surgery (GS) team and planned for surgery. The endocrinology team was involved for further evaluation. Upon questioning, the patient stated that he has been married for two and a half years and has fair libido and erectile function; however, he has no kids yet. Further, he addressed that he has been complaining of testicular swellings for a long time. Family history was remarkable for malignancy as his mother developed rectal cancer and his father was diagnosed with leukemia.

On examination, the patient was fully awake and communicating, with noted pigmented skin. Genital examination revealed small testicles with palpable hard swellings along with enlarged epididymis and penile shaft unremarkable with normal meatus. The patient admitted that he was diagnosed with adrenal disease during childhood and was prescribed steroids; nevertheless, he used them for a short time only.

The full hormonal profile besides tumor markers was requested (Table 2). Results were notable for primary adrenal insufficiency as well as elevated 17-hydroxyprogesterone level plus hyperprolactinemia. Testicular ultrasound showed bilateral testicular and epididymal lesions variable in echogenicity and vascularity. Pituitary fossa MRI showed no detectable sellar or suprasellar lesions.

**Table 2:** Hormonal profile and tumor marker results ACTH: Adrenocorticotropic hormone, AM: ante meridiem “morning”, DHEAS: dehydroepiandrosterone sulfate, TSH: Thyroid-Stimulating Hormone, T4: thyroxin 4, LH: Luteinizing hormone, FSH: Follicle-stimulating hormone, IGF-1: insulin-like growth factor 1, TV: total volume, PSA: Prostate-specific antigen, hCG: Human chorionic gonadotropin, AFP: Alpha fetoprotein, CEA: carcinoembryonic antigen, CA: cancer antigen.

Exam	Result	Reference Range
ACTH	119.4	7.2-63.3 pg/mL
Cortisol AM	44	102.1-535.2 nmol/L
DHEAS	0.57	3.8-13.10 umol/L
17-Hydroxyprogesterone	1110.7	0-6.66 nmol/L
TSH	2.98	0.35-4.94
Free T4	15.94	9-19 pmol/L
Prolactin	2748.90	mlU/L
Testosterone Total	5.10	nmol/L
LH	0.42	IU/L
FSH	1.53	IU/L
IGF-1	86	63-223
Renin	457.2	4.2-59.7 uIU/ml
Aldosterone	<3.7	<31 ng/dl
Aldosterone/Renin Ratio	Unable to calculate	-
Metanephrine 24 Urine	0.11	~1.62 umol/24h
Normetanephrines 24 Urine	1.64	~2.13 umol/24h
Urine TV	2600 ml	-
PSA	0.67	<4 ug/L
Beta hCG Quantitative	<2.3	<5 IU/L
AFP	<2	<8 ng/mL
CEA	1.7	<5 ng/mL
CA 19.9	4	<37 U/ml
CA 125	5	<35 U/ml

So, the impression was made which is the coexistence of giant AMs with adrenal insufficiency, which raised the possibility of CAH that is complicated by testicular adrenal rest tumors (TARTs) due to medication non-compliance. Oral glucocorticoid (hydrocortisone) was started in divided doses (10 mg AM + 20 mg PM). After careful Cardiology and Anesthesia teams' assessment and clearance, the patient underwent successful exploratory laparotomy plus bilateral adrenalectomy with coverage of intravenous (IV) glucocorticoid intraoperatively. The patient was shifted to the Intensive Care Unit (ICU) for monitoring; later after five days, he was transferred to the regular floor. Tapered IV glucocorticoid was shifted to oral, and the patient felt well and was hemodynamically stable and discharged home in good condition.

During follow-up in the clinic, the patient reported feeling dizzy while getting up from the seated position; otherwise, there were no other significant complaints. Abdomen on exam was soft, lax, and not tender, laparotomy wound was healing well with no signs of infection. New labs showed potassium of 4.9 and sodium of 138. Fludrocortisone 0.1 mg was initiated. Surgical pathology final reports revealed bilateral adrenal myelolipomas, with the right adrenal gland mass measuring 28x16x5 cm and weighing 1702 grams, and the left adrenal gland mass measuring 39x17x8 cm and weighing 4050 grams. The surgical margins were negative for tumor, and residual thin rims of adrenal tissue were identified. The patient was referred to Urology for infertility evaluation and management.

## Discussion

AMs are in the majority of cases unilateral and asymptomatic and often less than 4 cm in diameter [12]. On the other hand, they may become larger in size and also be bilateral, similar to our case. While myelolipomas are usually incidental findings, they may occasionally become symptomatic as a result of the mass effect, thus, causing pressure in the surrounding structures.

The pathogenesis of AMs is still uncertain, and one of the prevalent hypotheses suggests that they arise from the zona fasciculate of the adrenal cortex from metaplasia of undifferentiated stromal cells [13]. Prolonged stimulation of the adrenal cortex by high ACTH levels contributes to the development of myelolipomas [14].

The diagnosis of AMs is primarily determined through imaging techniques such as CT or MRI, with (better) or without contrast enhancement, which helps in identifying the macroscopic fat components present in the tumor [15]. These tumors generally exhibit a rounded shape predominantly consisting of macroscopic fat, but they also contain varying amounts of attenuating myeloid components that can appear in different patterns including a cloudy pattern, solid strands, or as a separate solid nodule within the fat [16].

In the presented case, the patient presented with symptoms of abdominal distention and obstruction. The final pathology report revealed bilateral AMs with the right adrenal mass measuring 28 cm and the left adrenal mass measuring 39 cm. These sizes are considered to be giant AMs. The largest such tumor documented in scientific literature had a diameter of 43 cm and weighed 9.6 kg, as reported by Allison and colleagues [17].

Although AMs do not contain any adrenal cortical or medullary components and are not hormonally active, coexistence of adrenocortical adenomas and hormone hypersecretion is reported [18]. Basic chemistry together with hormonal workup (including aldosterone renin ratio, metanephrines, cortisol post 1 mg dexamethasone, ACTH, and DHEAS) should be considered in patients with suspected coexisting adrenal adenoma and those with features of abnormal hormone production (such as CAH), rather all patients with myelolipomas.

Depending on the size, symptoms, and the functionality of the myelolipoma, the optimal treatment is decided. For asymptomatic AMs smaller than 4 cm surveillance seems to be enough as there is a little risk for complications; on the contrary, symptomatic, complicated, larger than 7 cm or hormonally active myelolipomas, should be surgically removed [12], like in our patient who did exploratory laparotomy plus bilateral adrenalectomy.

It is well known that uncontrolled replacement of glucocorticoids can lead to TARTs, which are benign ACTH-dependent tumors that occur in males with CAH [19] if left untreated can lead to the increase of the intratesticular pressure, impairs flow to the testis, and hinders outflow of semen; thus, TARTs can cause infertility by many mechanisms, like our patient who is married for two and half years and has no kids, also he admits that he was on “steroid” treatment during childhood but he is off since very long time. Adequate glucocorticoid therapy could result in the regression of these testicular tumors [20].

## Conclusions

In conclusion, this case emphasizes the importance of considering AMs as a potential diagnosis in patients with a history suggestive of CAH presenting with abdominal pressure symptoms. Early diagnosis and prompt surgical intervention are crucial in preventing complications associated with giant AMs, the diverse manifestations, and associations of AM, pointing to a necessity for comprehensive diagnostic approaches in conjunction with multidisciplinary team efforts. Further research studies are needed for better understanding of the pathogenesis and optimal management of AMs, particularly in patients with CAH.
